# *TERT-CLPTM1L* Rs401681 C>T Polymorphism Was Associated with a Decreased Risk of Esophageal Cancer in a Chinese Population

**DOI:** 10.1371/journal.pone.0100667

**Published:** 2014-07-09

**Authors:** Jun Yin, Liming Wang, Liang Zheng, Xu Wang, Yijun Shi, Aizhong Shao, Guowen Ding, Chao Liu, Suocheng Chen, Weifeng Tang, Haiyong Gu

**Affiliations:** 1 Department of Cardiothoracic Surgery, Affiliated People's Hospital of Jiangsu University, Zhenjiang, Jiangsu Province, China; 2 Cancer institute, Department of chemotherapy, People's Hospital Affiliated to Jiangsu University, Zhenjiang, Jiangsu Province, China; 3 Department of Cardiothoracic Surgery, The First People's Hospital of Changzhou and The Third Affiliated Hospital of Suzhou University, Changzhou, Jiangsu Province, China; Roswell Park Cancer Institute, United States of America

## Abstract

**Background:**

Esophageal cancer was the fifth most commonly diagnosed cancer and the fourth leading cause of cancer-related death in China in 2009. Esophageal squamous cell carcinoma (ESCC) accounts for more than 90 percent of esophageal cancers. Genetic factors probably play an important role in the ESCC carcinogenesis.

**Methods:**

We conducted a hospital based case-control study to evaluate functional *hTERT* rs2736098 G>A and *TERT-CLPTM1L* rs401681 C>T single nucleotide polymorphisms (SNPs) on the risk of ESCC. Six hundred and twenty-nine ESCC cases and 686 controls were recruited. Their genotypes were determined using the ligation detection reaction (LDR) method.

**Results:**

When the *TERT-CLPTM1L* rs401681 CC homozygote genotype was used as the reference group, the CT genotype was associated with a significantly decreased risk of ESCC (adjusted OR  = 0.74, 95% CI  = 0.58–0.94, *p* = 0.012); the CT/TT variants were associated with a 26% decreased risk of ESCC (adjusted OR  = 0.74, 95% CI  = 0.59–0.93, *P* = 0.009). The significantly decreased risk of ESCC associated with the *TERT-CLPTM1L* rs401681 C>T polymorphism was associated with male sex, young age (<63 years in our study) and alcohol consumption. No association between the *hTERT* rs2736098 G>A polymorphism and ESCC risk was observed.

**Conclusion:**

*TERT-CLPTM1L* rs401681 CT and CT/TT genotypes were associated with decreased risk of ESCC, particularly among men, young patients and those reported to be drinkers. However, our results are preliminary conclusions. Larger studies with more rigorous study designs are required to confirm the current findings.

## Introduction

Esophageal cancer was the fifth most commonly diagnosed cancer and the fourth leading cause of cancer-related death in China in 2009 [1]. Esophageal cancer is very aggressive and is associated with a poor prognosis [2]. Esophageal squamous cell carcinoma (ESCC) accounts for more than 90 percent of esophageal cancers [3]. Smoking and heavy drinking are major environmental risk factors for ESCC [4]. However, only a subset of individuals exposed to these environmental risk factors develops ESCC, suggesting that genetic factors, such as single nucleotide polymorphisms (SNPs), may also contribute to ESCC carcinogenesis.

Recently, several genome-wide association studies (GWAS) reported that common polymorphisms of Telomerase reverse transcriptase-cleft lip and palate transmembrane 1 like, CLPTM1L (*TERT-CLPTM1L*), which is located at locus 5p15.33, were associated with the risk of many types of cancer [5], [6]. The 5p15.33 locus, which is associated with telomerase function, contains two key genes: the *TERT* gene and the *CLPTM1L* gene. The *TERT-CLPTM1L* SNP, rs401681 (C>T, located in intron 13 of *CLPTM1L*, 27 kb from the *TERT* gene), is one of the most extensively studied SNPs. Two variants in 5p15 (rs401681 and rs2736098) are significantly associated with bladder cancer in individuals of European ancestry. These variants are in linkage disequilibrium (LD) with *CLPTM1L* and *TERT*, and both variants are also associated with basal cell carcinoma [6], lung cancer [7], glioma [8] and other tumors [6]. The *TERT-CLPTM1L* rs401681 C allele is also associated with shorter mean telomere length in lymphocytes [9]. Telomerase is expressed in stem cell lines and is inactive in somatic cells [10]. TERT is the reverse transcriptase catalytic subunit of telomerase, whose induction, together with telomerase activation, are critical steps during cellular immortalization and transformation [11]. Telomeres can become dysfunctional for a variety reasons, such as gradual shortening caused by incomplete replication of chromosomes, oxidative DNA damage or mutations in structural proteins, such as TERT [12]. Mutations in the coding regions of TERT can affect telomerase activity and telomere length, resulting in severe clinical phenotypes, including bone marrow failure syndromes and a substantial increase in cancer frequency [13].

The role of CLPTM1L was initially described in ovarian cancer cells, where overexpression of the gene induced apoptosis in cisplatin-sensitive cells [14]. CLPTM1L is also involved in mitochondrial apoptosis in normal cells, and was reported to be overexpressed in lung cancer cells [15]. The *CLPTM1L* gene may play a role in the apoptotic response. Overexpression of *CLPTM1L* mRNA has been observed in many cancer types including non-melanoma skin cancers [13]. Although the function of the *CLPTM1L* gene is largely unknown, studies have demonstrated that it may induce apoptosis. For example, CLPTM1L, as a predicted transmembrane protein, is upregulated in cisplatin-resistant ovarian cancer cell lines, and may be involved in the apoptotic response of cells to cisplatin-induced genotoxic stress. *CLPTM1L* variants are hypothesized to enhance the metabolic activation of reactive metabolites and/or the formation and persistence of DNA adducts [6]. Jiang et al. found that *TERT-CLPTM1L* rs401681 was a genetic variant associated with the risk of lung cancer [16].

The biological and pathological significance of hTERT and TERT-CLPTM1L suggests that functional genetic variations in the *hTERT* and *TERT-CLPTM1L* genes may contribute to the development of ESCC. Thus, the objective of this investigation was to evaluate the association between *hTERT* rs2736098 G>A and *TERT-CLPTM1L* rs401681 C>T polymorphisms and ESCC susceptibility in a hospital-based case-control study. We performed genotyping analyses of *hTERT* rs2736098 G>A and *TERT-CLPTM1L* rs401681 C>T SNPs in 629 ESCC cases and 686 controls in a Chinese population.

## Patients and Methods

### Ethical approval of the study protocol

This hospital-based case-control study was approved by the Review Board of Jiangsu University (Zhenjiang, China). We have complied with the World Medical Association Declaration of Helsinki regarding ethical conduct of research involving human subjects and/or animals [17]. All subjects provided written informed consent to be included in the study.

### Study subjects

629 subjects with esophageal cancer were consecutively recruited from the Affiliated People's Hospital of Jiangsu University and Affiliated Hospital of Jiangsu University (Zhenjiang, China) between October 2008 and December 2010. All cases of esophageal cancer were diagnosed as ESCC by pathologic means. The exclusion criteria were patients who previously had: cancer; any metastasized cancer; radiotherapy or chemotherapy. The 686 controls were patients without cancer frequency-matched to the cases with regard to age (±5 years) and sex recruited from the two hospitals mentioned above during the same time period. Most of the controls were admitted to the hospitals for the treatment of trauma.

Each subject was personally questioned by trained interviewers using a pre-tested questionnaire to obtain information on demographic data (e.g., age, sex) and related risk factors (including tobacco smoking and alcohol consumption). After the interview, 2-mL samples of venous blood were collected from each subject. Individuals who smoked one cigarette per day for >1 year were defined as “smokers”. Subjects who consumed ≥3 alcoholic drinks a week for >6 months were considered to be “alcohol drinkers”.

### Isolation of DNA and genotyping by ligation detection reaction and online SNP function prediction

Blood samples were collected from patients using Vacutainers and transferred to tubes lined with ethylenediamine tetra-acetic acid (EDTA). Genomic DNA was isolated from whole blood with the QIAamp DNA Blood Mini Kit (Qiagen, Berlin, Germany) [18]. The samples were genotyped using the ligation detection reaction (LDR) method with technical support from the Shanghai Biowing Applied Biotechnology Company as previously described [19]. SNPs were genotyped using the polymerase chain reaction (PCR)LDR assay by ABI 9600 system (Applied Biosystems, USA). The target DNA sequences were amplified by themultiplex PCR method. The common probe was labeled at the 3′end with 6-carboxy-fluorescein and phosphorylated at the 5′ end. LDR parameters were as follows: 94°C for 2 min, 20 cycles of 94°C for 30 s and 60°C for 3 min. After the LDR reaction, we mixed 1 µL LDR-reaction product with 1 µL ROX and 1 µL loading buffer. After that, the mixture was denatured at 95°C for 3 min and chilled in ice water immediately. The fluorescent products of LDR were differentiated by ABI sequencer 377 (Applied Biosystems, USA). For quality control, repeated analyses were done for 160 (12.17%) randomly selected samples with high DNA quality.

We used online predictive tool: http://www.regulomedb.org/and http://snpinfo.niehs.nih.gov/snpinfo/snpfunc.htm to predict *hTERT* rs2736098 G>A and *TERT-CLPTM1L* rs401681 C>T SNPs function.

### Statistical Analyses

Differences in the distributions of demographic characteristics, selected variables, and genotypes of the *hTERT* rs2736098 G>A and *TERT-CLPTM1L* rs401681 C>T variants between the cases and controls were evaluated using the *χ* ^2^ test. The associations between *hTERT* rs2736098 G>A and *TERT-CLPTM1L* rs401681 C>T genotypes and risk of ESCC were estimated by computing the ORs and their 95% CIs using logistic regression analyses for crude ORs and adjusted ORs when adjusting for age, sex, smoking and drinking status. The Hardy-Weinberg equilibrium (HWE) was tested by a goodness-of-fit *χ* ^2^ test to compare the observed genotype frequencies to the expected ones among the control subjects. All statistical analyses were performed with SAS 9.1.3 (SAS Institute, Cary, NC, USA).

## Results

### Characteristics of the study population

Characteristics of cases and controls included in the study are summarized in Table 1. The cases and controls appeared to be adequately matched on age and sex as suggested by the *χ* ^2^ tests (*P* = 0.541 and *P* = 0.185, respectively). As shown in Table 1, significant difference was detected on smoking status between the cases and the controls (*P*<0.001), and drinking rate was higher in ESCC patients than in control subjects (*P*<0.001). The primary information for two genotyped SNPs was in Table S1. For the *hTERT* rs2736098 G>A, the genotyping was successful in 600 (95.39%) ESCC cases and 651 (94.90%) controls in all 1315 samples, and for *TERT-CLPTM1L* rs401681 C>T, the genotyping was successful in 604 (96.03%) ESCC cases and 664 (96.78%) controls. The concordance rates of repeated analyses were 100%. Minor allele frequency (MAF) in our controls was similar to MAF for Chinese in database for all two SNPs (Table S1). The observed genotype frequencies for these two polymorphisms in the controls were all in HWE (Table S1).

**Table 1:** Distribution of selected demographic variables and risk factors in ESCC cases and controls.

	Cases (n = 629)		Controls (n = 686)		
Variable	n	(%)	n	(%)	*P* a
**Age (years)** mean ± SD	62.85 (±8.13)		62.58 (±7.89)		0.541
**Age (years)**					0.155
< 63	310	(49.28)	365	(53.21)	
≥ 63	319	(50.72)	321	(46.79)	
**Sex**					0.185
Male	444	(70.59)	461	(67.20)	
Female	185	(29.41)	225	(32.80)	
**Tobacco use**					**<0.001**
Never	355	(56.44)	499	(72.74)	
Ever	274	(43.56)	187	(27.26)	
**Alcohol use**					**<0.001**
Never	428	(68.04)	526	(76.68)	
Ever	201	(31.96)	160	(23.32)	

a Two-sided *χ* ^2^ test and student *t* test; Bold values are statistically significant (*P*<0.05).

### Associations between two polymorphisms and risk of ESCC

The genotype distributions of *hTERT* rs2736098 G>A and *TERT-CLPTM1L* rs401681 C>T in the cases and the controls are shown in Table 2. In the single locus analyses, the genotype frequencies of *TERT-CLPTM1L* rs401681 C>T were 47.68% (CC), 41.72% (CT), and 10.60% (TT) in the case patients and 40.06% (CC), 47.74% (CT), and 12.20% (TT) in the control subjects, and the difference was statistically significant (*P* = 0.024). When the *TERT-CLPTM1L* rs401681 C allele was used as the reference group, the T allele was associated with a significantly decreased risk for ESCC (T *vs* C: adjusted OR  = 0.81, 95% CI  = 0.69–0.96, *P* = 0.014). When the *TERT-CLPTM1L* rs401681 CC homozygote genotype was used as the reference group, the CT genotype was associated with a significantly decreased risk for ESCC (CT *vs* CC: adjusted OR  = 0.74, 95% CI  = 0.58–0.94, *P* = 0.012). When the *TERT-CLPTM1L* rs401681 CC homozygote genotype was used as the reference group, the TT genotype was not associated with the risk for ESCC (TT *vs* CC: adjusted OR  = 0.75, 95% CI  = 0.51–1.09, *P* = 0.126). In the dominant model, the *TERT-CLPTM1L* rs401681 CT/TT variants were associated with a 26% decreased risk of ESCC, compared with the *TERT-CLPTM1L* rs401681 CC genotype (adjusted OR  = 0.74, 95% CI  = 0.59–0.93, *P* = 0.009). In the recessive model, when the *TERT-CLPTM1L* rs401681 CC/CT genotypes were used as the reference group, the TT homozygote genotype was not associated with the risk for ESCC (adjusted OR  = 0.87, 95% CI  = 0.61–1.24, *P* = 0.447) (Table 2).

**Table 2:** Logistic regression analyses of associations between *hTERT* rs2736098 G>A and *TERT-CLPTM1L* rs401681 C>T polymorphisms and risk of ESCC.

	Cases (n = 629)		Controls (n = 686)						
Genotype	n	(%)	n		(%)	Crude OR (95%CI)	*P*	Adjusted OR a (95%CI)	*P*
*hTERT* rs2736098 G>A									
GG	245	(40.83)		270	(41.47)	1.00 (reference value)		1.00 (reference value)	
GA	277	(46.17)		306	(47.00)	1.00 (0.79–1.27)	0.984	1.01 (0.79–1.28)	0.970
AA	78	(13.00)		75	(11.52)	1.15 (0.80–1.65)	0.459	1.18 (0.82–1.71)	0.372
AA vs. GA vs. GG							0.727		
GA/AA	355	(59.17)		381	(58.53)	1.03 (0.82–1.29)	0.818	1.04 (0.83–1.31)	0.742
GG/GA	522	(87.00)		576	(88.48)	1.00 (reference value)		1.00 (reference value)	
AA	78	(13.00)		75	(11.52)	1.15 (0.82–1.61)	0.425	1.18 (0.84–1.67)	0.348
G allele	767	(63.92)		846	(64.98)	1.00 (reference value)		—	
A allele	433	(36.08)		456	(35.02)	1.05 (0.89–1.23)	0.580	—	—
*TERT-CLPTM1L* rs401681 C>T									
CC	288	(47.68)		266	(40.06)	1.00 (reference value)		1.00 (reference value)	
CT	252	(41.72)		317	(47.74)	**0.73 (0.58–0.93)**	**0.010**	**0.74 (0.58–0.94)**	**0.012**
TT	64	(10.60)		81	(12.20)	0.73 (0.51–1.05)	0.093	0.75 (0.51–1.09)	0.126
TT vs. CT vs. CC							**0.024**		
CT/TT	316	(52.32)		398	(59.94)	**0.73 (0.59–0.92)**	**0.006**	**0.74 (0.59–0.93)**	**0.009**
CC/CT	540	(89.40)		583	(87.80)	1.00 (reference value)		1.00 (reference value)	
TT	64	(10.60)		81	(12.20)	0.85 (0.60–1.21)	0.371	0.87 (0.61–1.24)	0.447
C allele	828	(68.54)		849	(63.93)	1.00 (reference value)		—	
T allele	380	(31.46)		479	(36.07)	**0.81 (0.69–0.96)**	**0.014**	—	—

a Adjusted for age, sex, smoking and drinking status; Bold values are statistically significant (*P*<0.05).

*hTERT* rs2736098 G>A was not showed a significant difference in the genotype distributions between cases and controls (*P* = 0.727). Logistic regression analyses revealed that the *hTERT* rs2736098 G>A polymorphism was not associated with the risk of ESCC (Table 2).

Using Power and Sample Size Calculation (PS, version 3.0, 2009, http://biostat.mc.vanderbilt.edu/twiki/bin/view/Main/PowerSampleSize) and considering *TERT-CLPTM1L* rs401681 C>T mutant alleles in the control group, ORs, ESCC samples and control samples, the power of our analysis (α = 0.05) was 0.708 in 604 ESCC cases and 664 controls, with an OR of 0.74.

### Stratification analyses on the TERT-CLPTM1L rs401681 C>T polymorphisms and the risk of ESCC

To evaluate the effects of *TERT-CLPTM1L* rs401681 C>T genotypes on ESCC risk according to different age, sex, smoking and alcohol drinking status; we performed the stratification analyses (Table 3). A significantly decreased risk of ESCC associated with the *TERT-CLPTM1L* rs401681 C>T polymorphism was evident among male patients (CT *vs* CC: *P* = 0.0003; CT/TT *vs* CC: *P* = 0.0002), younger patients (<63 years in our study) (CT/TT *vs* CC: *P* = 0.049) and patients who were in drinking status (CT *vs* CC: *P* = 0.012; CT/TT *vs* CC: *P* = 0.016) (Table 3).

**Table 3:** Stratified analyses between *TERT-CLPTM1L* rs401681 C>T polymorphism and ESCC risk by sex, age, smoking status and alcohol consumption.

	*TERT-CLPTM1L* rs401681 C>T (case/control) a				Adjusted OR b (95% CI); *P*; *P_h_* c				
Variable	CC	CT	TT	CT/TT	CC	CT	TT	CT/TT	TT vs. (CT/CC)
Sex									
Male	219/172	163/216	45/56	208/272	1.00 (reference value)	**0.59 (0.44–0.78); ** ***P*** **: 0.0003**; ***P*** **_h_:0.007**	0.64 (0.41–1.00); *P*: 0.052; *P*_h_:0.227	**0.60 (0.45–0.79); ** ***P*** **: 0.0002**; ***P*** **_h_:0.007**	0.83 (0.54–1.27); *P*: 0.397; *P*_h_:0.719
Female	69/94	89/101	19/25	108/126	1.00 (reference value)	1.24 (0.81–1.89); *P*: 0.330; ***P*** **_h_:0.007**	1.11 (0.56–2.20); *P*: 0.757; *P*_h_:0.227	1.21 (0.81–1.82); *P*: 0.355; ***P*** **_h_:0.007**	0.99 (0.52–1.88); *P*: 0.979; *P*_h_:0.719
Age									
<63	147/142	119/158	31/49	150/207	1.00 (reference value)	0.75 (0.53–1.06); *P*: 0.099; *P*_h_:0.963	0.64 (0.38–1.08); *P*: 0.091; *P*_h_:0.297	**0.72 (0.52–1.00); ** ***P*** **: 0.049**; *P*_h_:0.699	0.73 (0.45–1.21); *P*: 0.221; *P*_h_:0.264
≥63	141/124	133/159	33/32	166/191	1.00 (reference value)	0.73 (0.52–1.03); *P*: 0.071; *P*_h_:0.963	0.93 (0.54–1.60); *P*: 0.790; *P*_h_:0.297	0.77 (0.56–1.06); *P*: 0.103; *P*_h_:0.699	1.09 (0.65–1.83); *P*: 0.739; *P*_h_:0.264
Smoking status									
Never	158/194	148/228	36/62	184/290	1.00 (reference value)	0.79 (0.59–1.07); *P*: 0.124; *P*_h_:0.414	0.73 (0.46–1.17); *P*: 0.189; *P*_h_:0.739	0.78 (0.59–1.03); *P*: 0.083; *P*_h_:0.562	0.82 (0.53–1.28); *P*: 0.391; *P*_h_:0.540
Ever	130/72	104/89	28/19	132/108	1.00 (reference value)	0.68 (0.45–1.03); *P*: 0.069; *P*_h_:0.414	0.82 (0.42–1.59); *P*: 0.556; *P*_h_:0.739	0.71 (0.48–1.05); *P*: 0.082; *P*_h_:0.562	0.99 (0.53–1.87); *P*: 0.980; *P*_h_:0.540
Alcohol consumption									
Never	189/209	176/236	44/65	220/301	1.00 (reference value)	0.84 (0.63–1.12); *P*: 0.231; *P*_h_:0.118	0.78 (0.50–1.21); *P*: 0.262; *P*_h_:0.928	0.83 (0.63–1.08); *P*: 0.167; *P*_h_:0.175	0.85 (0.56–1.29); *P*: 0.439; *P*_h_:0.665
Ever	99/57	76/81	20/16	96/97	1.00 (reference value)	**0.55 (0.35–0.88); ** ***P*** **: 0.012**; *P*_h_:0.118	0.74 (0.35–1.56); *P*: 0.421; *P*_h_:0.928	**0.58 (0.37–0.90); ** ***P*** **: 0.016**; *P*_h_:0.175	1.00 (0.49–2.03); *P*: 0.996; *P*_h_:0.665

a The genotyping was successful in 600 (95.4%) ESCC cases, and 651 (94.9%) controls for *TERT-CLPTM1L* rs401681 C>T;

b Adjusted for age, sex, smoking status and alcohol consumption (besides stratified factors accordingly) in a logistic regression model;

c *P_h_* for heterogeneity; Bold values are statistically significant (*P or P_h_*<0.05).

## Discussion

In this hospital-based case-control study, we investigated the association of the *hTERT* rs2736098 G>A and *TERT-CLPTM1L* rs401681 C>T SNPs with the risk of ESCC in a Chinese population. Multivariate logistic analysis revealed that the *TERT-CLPTM1L* rs401681 CT and CT/TT genotypes were associated with decreased risk of ESCC, whereas no significant association between the *hTERT* rs2736098 G>A polymorphism and the risk of ESCC was observed. A significantly decreased risk of ESCC associated with the *TERT-CLPTM1L* rs401681 C>T polymorphism, particularly among men, young patients and those reported to be drinkers.

The function of CLPTM1L and its role in tumorigenesis is largely unknown. However, a recent study reported that CLPTM1L was a commonly overexpressed anti-apoptotic factor in lung cancer [15]. This suggested an inhibitory role in genotoxic stress-induced apoptosis, and identified CLPTM1L as an important factor affecting the survival of DNA damaged tumor cells and potentially lung cancer susceptibility [20].

The *CLPTM1L* gene is upregulated in cisplatin-resistant cell lines, and is linked to cisplatin-induced apoptosis; furthermore, over-expression of *CLPTM1L* mRNA has been observed in many cancers [6], [14], [21], [22]. Variants at this locus are hypothesized to regulate telomere length and be associated with multiple malignancies, including cancers of the lung, prostate, urinary bladder, cervix and pancreas. Rs401681 is located in intron 13 of *CLPTM1L* at 5p15.33, and it is one of the most studied SNPs. Although little is known about the function of this SNP, our bioinformatics analysis indicated that it might affect transcription regulation and further affect the expression of the gene. To show that these alterations can indeed contribute to cancer properties, invitro validation studies with specific invitro cell lines of ESCC that harbor these genetic alterations are warrented. Such as cell cultures, transient transfections, luciferase assay, electrophoretic mobility shift assays, Western blot analysis, reverse transcriptase PCR, chromatin immunoprecipitation assays and quantitative Real-Time PCR.

Several studies addressing the association between the *CLPTM1L* rs401681 polymorphism and cancer have been published, with inconsistent results [6], [15], [19], [23], [24], [25]. An association study that included 2,396 lung cancer cases and 3,001 controls showed that the *CLPTM1L* T allele was associated with a significantly decreased risk of lung cancer [15]. Nan and collaborators observed a suggestive positive relationship between the rs401681 C allele and shorter relative telomere length [7]. Rafnar et al. suggested that the rs401681 C allele might be associated with the acceleration of the gradual shortening of telomeres with age [6]. Possible links between shorter telomeres and decreased risk of melanoma have been reported. This could be attributed to the shorter replicative lifespan of melanocytes conferred by a shorter telomere length, which provides a more stringent barrier to unlimited cell division. A decreased risk of melanoma might also be associated with the reduction of nevi size and count in individuals with shorter telomeres. Rs401681 is also associated with the risk of pancreatic cancer, as shown by the presence of chromosome ends lacking telomeric repeat sequences in this cancer [26]. Jiang et al. found that *TERT-CLPTM1L* rs401681 T allele was associated with decreased risk of lung cancer [16]. And in ESCC cohort, the trend of *TERT-CLPTM1L* rs401681 T allele is protective but not reach significant (OR  = 0.935, 95% CI  = 0.800–1.093 in additive model) [16], indicating necessary for replications in other cohors.

The frequencies of genetic polymorphisms often vary between ethnic groups. In the present Chinese study, the allele frequency of *TERT-CLPTM1L* rs401681 T was 0.361 in 686 control subjects, which is consistent with the values reported in the SNP database for the Chinese Han (0.305) and Japanese populations (0.343); however, the frequency was lower than that of a Sub-Saharan African (0.642) population (http://www.ncbi.nlm.nih.gov/SNP).

This case-control study had several limitations. First, the patients and controls were enrolled from hospitals and may therefore not be representative of the general population; the information familial cancer history of the cases and controls was not obtained, this inherent bias may have resulted in spurious findings. Second, the polymorphisms investigated in our study were based on functional considerations and may not provide a comprehensive view of the genetic variability of *TERT-CLPTM1L*, such as rs402710 and rs2736100 et al. Further studies are needed to clarify the genetic mechanism of esophageal carcinogenesis by fine-mapping the susceptibility region of the variants. Third, the statistical power of our study was limited because of the moderate sample size and absence of a validation cohort. Larger, well-designed studies are warranted to confirm the associations observed in the present study. Finally, we did not obtain detailed information on cancer metastasis and survival, which further restricted the analysis of the roles of the *hTERT* rs2736098 G>A and *TERT-CLPTM1L* rs401681 C>T polymorphisms in ESCC progression and prognosis.

In conclusion, our study provides strong evidence that the functional *TERT-CLPTM1L* rs401681 C>T polymorphism may contribute to the risk of ESCC. However, the exact functional relevance of the *CLPTM1L* rs401681 SNP remains unclear. It may be in strong LD with other potential functional or causal SNPs, contributing to the risk of ESCC. Additional, larger studies and in vitro or tissue-specific biological characterization are required to confirm the current preliminary findings.

## Supporting Information

Table S1 **Primary information for** ***hTERT*** **rs2736098 G>A and** ***TERT-CLPTM1L*** **rs401681 C>T polymorphisms.**
(DOCX)

Data S1 **Data of** ***hTERT*** **rs2736098 G>A and** ***TERT-CLPTM1L*** **rs401681 C>T polymorphisms.**
(SAV)
