# Supplementary material for: TERT-CLPTM1L Rs401681 C>T Polymorphism Was Associated with a Decreased Risk of Esophageal Cancer in a Chinese Population
Source: PLoS One. 2014 Jul 9;9(7):e100667. doi: 10.1371/journal.pone.0100667 (PMC4089909; doi:10.1371/journal.pone.0100667)
Supplement: Table S1 — Primary information for hTERT rs2736098 G>A and TERT-CLPTM1L rs401681 C>T polymorphisms. (DOCX) [file pone.0100667.s001.docx]

**Table S1** Primary information for *hTERT* rs2736098 G>A and *TERT-CLPTM1L* rs401681 C>T polymorphisms

| Genotyped SNPs | *hTERT*  rs2736098 G>A | *TERT-CLPTM1L* rs401681 C>T |
| --- | --- | --- |
| Chromosome | 5 | 5 |
| Function | synonymous | intron |
| Chr Pos (Genome Build 36.3) | [1347086](http://www.ncbi.nlm.nih.gov/sites/nuccore/NC_000005.8?report=graph&v=1346586:1347586&content=5&m=1347086!&mn=rs2736098&dispmax=1&currpage=1) | [1375087](http://www.ncbi.nlm.nih.gov/sites/nuccore/NC_000005.8?report=graph&v=1374587:1375587&content=5&m=1375087!&mn=rs401681&dispmax=1&currpage=1) |
| Regulome DB Score^a^ | 5 | 5 |
| MAF^b^ for Chinese in database | 0.411 | 0.305 |
| MAF in our controls (n = 686) | 0.350 | 0.361 |
| *P* value for HWE^c^  test in our controls | 0.403 | 0.365 |
| Genotyping method^d^ | LDR | LDR |
| % Genotyping value | 95.13% | 96.43% |

^a^ http://www.regulomedb.org/;

^b^ MAF: minor allele frequency;

^c^ HWE: Hardy–Weinberg equilibrium;

^d^ LDR: ligation detection reaction.
